# Mercy of Prompt Surgery

**Published:** 1873-12

**Authors:** Theo A. McGraw

**Affiliations:** Professor of Surgery in Detroit Medical College


### THE MERCY OF PROMPT SURGERY.

BY THEO A. McGRAW, M.D.,
Professor of Surgery in Detroit Medical College.

The unreasoning dread with which the public regard all surgical operations seems to have extended itself to certain members of the medical profession. There are surgeons who apparently think it necessary to apologize for resorting to the knife, and they are apt to do so in the stereotyped phrase, "that they had first tried all other imaginable remedies without success." The conviction, implied in that phrase, that no operation is justifiable which could by any possible means be avoided, is, I hold, utterly childish and illogical. Surgical operations should be resorted to whenever and wherever they seem to the surgeon to offer the *best* and most certain means of relief, and to delay them for the sake of trying inefficient and worthless remedies is frequently to subject the patient to irreparable injury. It is my own belief that timidity and cowardly inaction have caused more deaths in the practice of surgery than can be attributed to all other forms of surgical malpractice. For, contrary to the popular conviction, the prevailing tendency among surgeons is *not* towards recklessness, but towards over-caution, and it occurs frequently to even the boldest operators to regret opportunities for cure which have been suffered to slip away, while practitioner and patient were living in expectancy or dallying with inefficacious remedies. Marked examples of this characteristic of our profession were to be met with in the army during the late war. The majority of surgeons seemed to be quite averse to operating, and it was often difficult to find a sufficient number to do the work necessary after a battle. Conservative surgery was carried in many instances to the most unwarrantable extent, and hundreds within my own experience lost their lives by the delay of operations which ought to have been performed on the field.

Very provoking instances of this timidity and tendency to postpone operative procedures occurred in the treatment of secondary hemorrhage. Every practical surgeon knows that an artery of the size of the radial, for example, if wounded, should be tied, or, at any rate, treated by some method equally efficient with the ligature. But the ligation of wounded arteries at the bleeding point is often one of the most difficult operations in surgery. Many surgeons, therefore, were accustomed to delay it as long as possible, and to control the hemorrhage in the meantime by means of styptics and compresses. The result was always the same. The bleeding temporarily checked, invariably recurred, and ligation had to be practiced at a later period, after the tissues had become inflamed and softened; when, in fact, everything had altered for the worse. I have known this to happen after wounds of the radial and ulnar arteries, of the palmar arches and the tibial arteries, and always to the injury of the patient. One or two examples of this kind have come within my knowledge as occurring in civil practice since the war. In one case, a steward on one of the upper lake steamers had the misfortune to thrust his hand and forearm through a pane of glass, cutting the radial artery about an inch above the wrist. A physician, who chanced to be aboard, applied a compress. Two or three days afterwards he arrived in the city and had secondary hemorrhage, the compress having been loosened. The practitioners who attended him re-adjusted the bandage, with the further application of liq. ferri persulph. Twice again the hemorrhage recurred, the last time during the night. I was then sent for, found and tied the artery without difficulty, above and below the wound, and the patient recovered without further trouble. Now, it cannot be doubted that hemorrhage from even large arteries will, occasionally, cease permanently under systematic compression, and if the rule which some practitioners advocate were to be adopted, that operative procedures should be postponed until the last, it should apply as well in cases of arterial hemorrhage as of stricture of the urethra, or the extirpation of cancerous tumors. In respect to bleeding from arteries, however, the profession are fortunately united, and surgeons, who do not follow the rule of ligation, would usually find it difficult to defend their practice.

In the opening of abscesses and cavities containing effusions of venous blood, surgeons, who are so disposed, will find abundant opportunity to follow the counsels of timidity, to the injury of their patients. The most common examples of this sort are furnished by patients who have suffered from whitlows. The majority of practitioners content themselves with the free use of poultices and a very small prick with the point of a bistoury. The weeks of anguish which such patients have endured in order to recover finally with stiff and useless fingers, attest the value of timid counsels. How many a finger has been lost which might have been saved by one good, deep, free incision! Abscesses of the neck and behind the pharynx frequently do great damage while the surgeon is hesitating about the diagnosis or treatment of the case. In one case of post-pharyngeal abscess which came within my knowledge, the nature of the disease was not recognized until the vital powers had become utterly exhausted. The patient, a young lady, died a few hours after the pus had been evacuated. In other cases of abscess of the neck I have known the pus to sink deep among the tissues, and to endanger the life of the patient, while a timid surgeon was letting "I dare not" wait upon "I would."

It is one of the most astounding and yet common experiences to find practitioners passing day after day in the treatment of such cases in all the agonies of doubt, when a small puncture with an exploring needle, or the needle of a hypodermic syringe, which would cause little pain and no danger, would clear up all doubtful points, and make the duty of the surgeon perfectly apparent.

A very frequent cause of death after gunshot and other wounds is the extravasation and decomposition of venous blood in cavities formed among the tissues. Such extravasations may be diagnosticated by the great discoloration of the skin in the immediate neighborhood. They demand no treatment unless the patient should show signs of septic poisoning, and *then* the treatment should be prompt and effectual. Free incisions should be made, the decomposing blood evacuated, and the cavities frequently syringed out with antiseptic fluids. Under this treatment, I have frequently seen patients, who had become jaundiced and delirious from septic poisoning, slowly recover. One of my sadder recollections is of a patient in the army who showed symptoms of septicæmia after gunshot wound of the thigh. He died under expectant and improper treatment. A *post-mortem* examination revealed a large cavity near the femoral vein, in which lay the bullet and a large quantity of fetid, black, half-coagulated blood. I have little doubt but that prompt and bold surgery would in this case have saved a valuable life. Diseases of the rectum and genito-urinary organs, owing perhaps to their disagreeable character, are too apt to drag along under inefficient treatment until they become chronic and intractable. I have, in a paper upon strictures of the urethra, already stated the objections to the tedious process of cure by means of bougies in certain cases. I have little doubt that the inflammations of the bladder and kidneys, which are so common in cases of old strictures, are due, in a measure, to the irritation and mechanical injuries which those organs suffer by the constant presence of an obstacle to the exit of urine. Prompt operative measures will, therefore, notwithstanding their own peculiar dangers, have this advantage over the process of slow dilatation, that they give immediate and complete relief to the bladder. When the disease is present in subjects who are impatient of slow methods of cure, or whose occupation is such as to forbid them, there can, to my mind, be no doubt of the propriety either of rapid dilatation or urethrotomy. Delay in treatment is, however, blamable in many others of this class of disorders besides stricture. Even the simple matter of passing a catheter is often postponed until it is unavoidable. Thus, for example, in the partial retention of urine from enlarged prostate, many secondary troubles arise, which might be avoided if only the surgeon would early and often empty the bladder completely with a catheter.

Sir Henry Thompson complains that surgeons allow the most favorable time for operating for stone to pass over through neglect in making a correct diagnosis, or timidity in the treatment of the case. Thus stones, which when small could have been crushed easily and without danger, are allowed to increase in size, and to continue in the bladder until they cause disease of the kidneys, and endanger the life of the patient. Michigan does not give her surgeons many opportunities for studying the nature of calculus, and my own experience in this respect is very limited, but it is fair to speak of it in this connection as one of the maladies in which timidity and inaction are injurious, and, it may be, disastrous to the patient. Fissure of the anus is a small matter, easily cured by one of the simplest of operations, and yet it is surprising how long patients are allowed to suffer untold agonies, while their doctor is experimenting with useless salves and suppositories. The operation for fistula in ano, too, is so simple and devoid of danger in uncomplicated cases, that one need hardly desire more satisfactory therapeutics, and yet there are surgeons who never cut the simplest fistula until they have racked their brains in vain for a method of cure which would not be open to the terrible objection of belonging to operative surgery.

I will speak of one more class of cases, and then have done. The labors of Virchow and others have thrown, in late years, wonderful light on the growth of morbid tumors. Of the benign tumors I have, in this connection, nothing to say. Without going into details as regards our knowledge of tumors of malignant or semi-malignant character, I may say that their growth and history seems to be marked by the following characteristics: They have their origin apparently at one point, and thence infect the system. The process of infection is carried on, first, through local contact and the forces of osmosis; second, through the blood vessels; and, third, through the lymphatics. The semi-malignant differ from the malignant tumors in not infecting the lymphatics. and in recurring more locally. Certain forms of sarcomatous tumors, too, seem to acquire their malignancy with age, being, in the earlier years of their growth, apparently innocuous. We have every reason to believe, from our present knowledge of the history of these cases, that even cancer might be cured, could its presence only be recognized in the earliest stage of its existence, and thus allow of its thorough extirpation while still limited to a small area.

If our pathology is correct, it would follow, logically, that all doubtful tumors should be extirpated thoroughly at a very early stage of their growth. The operation for the removal of a small lump in the breast, or side, or thigh, is so insignificant as to scarcely at all endanger the life of the patient, while the risk of death from cancer is terrible to contemplate, and is to be dreaded in all cases of doubtful growths or indurations which will not in the course of a few months yield to treatment. In my opinion, therefore, it becomes the duty of the physician, who has treated such lumps and indurations for a certain period without success, to urge upon his patient the operation for their removal. I, myself, never allow such a tumor to continue more than a very few months without counseling a resort to the knife. It is true, that in this way surgeons run the risk of removing innocent growths, and possibly inflammatory swellings, without urgent necessity; but how can the practical physician, for a moment, compare such risk with that which a patient runs before whom looms in the future the dreadful possibility of cancer? And yet it is the almost universal custom with general practitioners, who are first consulted about such cases, to advise delay, though delay may bring in its train incurable disease and death. I believe that it is in this class of cases especially that practical surgeons should urge upon the profession the dangers of inaction, and the pressing necessity of prompt surgery. The knife should not, gentlemen, be postponed in cases where it is needed, until the patient presses it upon the practitioner, but the surgeon should, in duty to humanity, firmly recommend its use, whenever and wherever it is the best instrument with which to effect the cure of the disease.—*Detroit Review of Medicine and Pharmacy.*

